# Surgicel plug technique versus endoclose technique for port-site closure post minimally invasive (robotic and laparoscopic) surgeries: Retrospective cohort study

**DOI:** 10.15537/smj.2023.44.4.20220899

**Published:** 2023-04

**Authors:** Maher S. Moazin, Abdulrahman M. Alsuwailim, Mosab M. Botaiban, Abdulaziz Y. Alabdulrahman, Abdullah Alfakhri, Naif Aldhaam,, Muhammad Abukhater, Arjmand Reyaz, Mohammed Y. Alessa

**Affiliations:** *From the Department of Urology (Moazin, Alfakhri, Aldhaam), and form the General Surgery Department (Abukhater, Reyaz), King Fahad Medical City, Riyadh; from the Urology Department (Alsuwailim), Almoosa Specialist Hospital; from the Internal Medicine Department (Botaiban, Alabdulrahman), and from the Surgery Department (Alessa), King Faisal University, Al Hasa, Kingdom of Saudi Arabia.*

**Keywords:** minimally invasive, port site, laparoscopic, robotic, Surgicel plug, endoclose

## Abstract

**Objectives:**

To compares the endoclose technique (ET) techniques and surgicel plug technique (SPT) in terms of port-site related complications. Minimally invasive surgeries (MIS) are widely performed nowadays, nonetheless, port-site closure technique plays a role in the prevention of port-site related complications.

**Methods:**

This retrospective study was carried out at general surgery and urology departments of King Fahad Medical City, Saudi Arabia. Variables that were collected include age, gender, height, weight, body mass index, co-morbidities, type and date of surgery, intraoperative visceral injury or bleeding, technique cost, and port-site post-operative complications (hernia, infection, bleeding, dehiscence, and hypertrophic scarring). Data was collected from electronic medical records. Patients included are whom underwent any minimally invasive procedure from the beginning of 2014 until the end of September 2020. Follow up period was at least for 2 years.

**Results:**

We analyzed 397 patients. Surgicel plug technique was more of having hernia (2.3%) than of ET (0%). While ET was more on infection (0.9%) than in SPT, but no significant difference being observed (*p*=0.064).

**Conclusion:**

There is no significant difference between the ET and the novel SPT in terms of port-site related complications.

**M**inimally invasive surgeries (MIS) are widely performed nowadays by their 2 means, robotic and laparoscopic. Port-site incisions in MIS may result in complications such as hernia, bleeding, infection, emphysema, visceral injury, or hypertrophic scarring.^1^ Multiple techniques have been invented over the past decades that uses different approaches and devices for port-sites closure; complications at port-sites post-operatively are varied among those techniques including hernia, bleeding, infection, dehiscence and hypertrophic scar.^2-5^ Endoclose technique is one of the widely performed techniques worldwide that is Fast, straightforward, and result in low complication rate.^6-9^ Surgicel material is an absorbable hemostatic agent that is commonly used to stop bleeding.^10^ There are different port-site closure techniques that had used recently hemostatic agents and succeeded to close the ports with low rate of complication.^11-14^ A comparative study showed the superiority of Surgicel plug over closure with Vicryl suture in terms of decreasing the overall cost, operative time, and iatrogenic bowel injuries caused by a closure device.^11^ Recently, a novel technique published which used a hemostatic agent (Surgicel) for port-site closure by plugging the ports; it is distinguished by its ease, fast to perform, low cost, effective in obese patients, lowers the tendency of post-operative port-site bleeding, does not need any device or needle, therefore, avoids the risk of injury to abdominal viscera and abdominal wall vessels.^13^ There is a lack in comparative studies with this novel technique, therefore, we compared between the novel Surgicel plug technique with the endoclose technique in terms of developing intraoperative or postoperative complications.

## Methods

This retrospective cohort study was carried out at General Surgery and Urology Departments of King Fahad Medical City, Riyadh, Saudi Arabia. Both techniques were used for trocar sizes 8 mm and more. Bariatric surgeons used the endoclose technique, whereas the urologists used the Surgicel plug technique. Data was collected from electronic medical records. Inclusion criteria are patients who underwent any minimally invasive (laparoscopic or robotic) procedure from the beginning of 2014 until the end of September 2020. Follow up period was at least for 2 years. Variables that were collected include: age, gender, height, weight, body mass index (BMI), comorbidities, type and date of surgery, intraoperative visceral injury or bleeding, and port-site post-operative complications (hernia, infection, bleeding, dehiscence, and hypertrophic scar). The exclusion criteria: patients whom their minimally invasive procedures had converted to open were excluded from the study. The hemostatic agent used is Surgicel^®^ (Ethicon, Somerville, NJ, US). Collected data was put in a specific secured excel file.

### Statistical analysis

Descriptive statistics were presented as numbers and percentages. The comparison between Surgicel plug technique and Endoclose technique in regard to the different characteristics of the patients had been carried out using Chi-square test. Generated significant results were then placed into multivariate regression model to determine the independent significant predictor associated with Endoclose technique where the odds ratio as well as 95% confidence interval were also being reported. A *p*-value of 0.05 was considered statistically significant. The data analyses were performed using IBM SPSS for Windows, version 26 (IBM Corp., Armonk, NY, USA).

### The novel Surgicel plug technique description

After peritoneum deflated post a laparoscopic or robotic procedure, the hemostatic agent (Surgicel) was inserted under scope guidance through the trocar opening and fed until half of the Surgicel passed inside. Then the trocar was removed leaving the hemostatic agent in place. After that, the outer half of the Surgicel was cut. The small remnant outer part was dipped just under the level of skin level. For the scope trocar, the Surgicel was placed blindly. At the end, the skin was closed with a stapler or with 4.0 monocryl suture in a subcuticular manner.

## Results

We analyzed 397 patients who underwent robotic or laparoscopic surgery. The highest number of surgery performed was sleeve gastrectomy (35.3%), followed by nephrectomy (25.4%). As Table 1 shows, the most common age group was 31-40 years old (25.9%) with nearly 60% were females. With regards to BMI level, majority of the patients were obese (67.5%) with BMI >30.

**Table 1:** - Basic demographic characteristics of the patients (N=397).

Study data	n (%)
***Age group in years***	
<18 years	30 (07.6)
18–30 years	89 (22.4)
31–40 years	103 (25.9)
41–50 years	75 (18.9)
51–60 years	55 (13.9)
>60 years	45 (11.3)
***Gender***	
Male	165 (41.6)
Female	232 (58.4)
***Body mass index***	
Underweight	13 (03.3)
Normal	51 (12.8)
Overweight	65 (16.4)
Obese	268 (67.5)

Table 2 described the surgical history, chronic disease and complication of the patients after surgery. It is observed that post-operative complication had been reported by 6 patients including: port-site hernia (4 cases) and wound infection (2 cases). The prevalence of patients with previous abdominal surgery was 24.9%. The proportion of patients with associated diabetes was 27%, cancer 4.5%, hypertension 27%, and coagulopathy 2%. Table 3 shows that most patients in both groups were overweight or obese, therefore, more susceptible to develop hernia.

**Table 2:** - Surgical history, chronic diseases and complication of the patients after surgery (N=397).

Variables	n (%)
***Follow up results***	
No complication	388 (97.7)
Hernia	04 (01.0)
Infection	02 (0.5)
***Patients had previous abdominal surgery***	
Yes	99 (24.9)
No	298 (75.1)
***Diabetes***	
Yes	107 (27.0)
No	290 (73.0)
***Hypertension***	
Yes	107 (27.0)
No	290 (73.0)
***Cancer***	
Yes	18 (04.5)
No	379 (95.5)
***Coagulopathy***	
Yes	04 (01.0)
No	393 (99.0)
***Surgical technique***	
Surgical plug technique	176 (44.3)
Endoclose technique	221 (55.7)

**Table 3:** - Comparison between Surgicel plug and Endoclose techniques in regards to the basic demographic and other related characteristics of the patients (N=397).

Factor	Surgical technique		*P*-value*
	Surgicel plug (n=176)	Endoclose (n=221)	
***Age group in years***			
≤40 years	82 (46.6)	140 (63.3)	0.001**
>40 years	94 (53.4)	81 (36.7)	
**Gender**			
Male	85 (48.3)	79 (35.7)	0.012 **
Female	91 (51.7)	142 (64.3)	
***Body mass index level***			
Normal or underweight	15 (08.5)	49 (22.2)	<0.001 **
Overweight or obese	161 (91.5)	172 (77.8)	
***Previous abdominal surgery***			
Yes	26 (14.8)	73 (33.0)	<0.001 **
No	150 (85.2)	148 (67.0)	
***Diabetes***			
Yes	39 (22.2)	68 (30.8)	0.055
No	137 (77.8)	153 (69.2)	
***Hypertension***			
Yes	46 (26.1)	61 (27.6)	0.630
No	130 (73.9)	159 (71.9)	
***Cancer***			
Yes	15 (08.5)	03 (01.4)	0.001 **
No	161 (91.5)	218 (98.6)	
***Follow up complication***			
No complication	170 (96.6)	218 (98.6)	0.064
Port-site hernia	04 (02.3)	0	
Wound infection	0	02 (0.90%)	

Values are presented as number and percentages (%). *P-value has been calculated using Chi-square test. **Significant at *p*<0.05 level.

In comparison between the surgical techniques, Table 4 shows different complications and the possible associated factors. It can be observed that Surgicel plug technique was more of having hernia (2.3%) while endoclose technique was more on infection (0.9%), but no significant difference being observed (*p*=0.064). We also observed that hypertension was associated with 2 hernia cases, diabetes was associated with 1 hernia case, and 1 hernia case had previous abdominal surgery (pyeloplasty). Port-site hernia development timeline among the 4 cases was between 4-18 months. All patients who develop hernia were clinically obese (BMI >30).

**Table 4:** - Associated risk factors of complications.

Factor	Surgicel plug	Endoclose	Coagulopathy	Diabetes	Hypertension	Previous abdominal surgery	Cancer
Hernia	4 (02.3)	0	0	1 (25.0)	02 (50.0)	1 (Pyeloplasty)	0
Infection	0	02 (0.9)	0	0	0	0	0

## Discussion

The current study was carried out to determine the efficacy and safety differences between the novel Surgicel plug technique and the endoclose technique by Carter Thomason (CT) device. The results showed that the Surgicel plug technique resulted in more hernia (2.3%) cases than Endoclose technique (0%). However, in terms of infection, the endoclose technique resulted in more rate of infection (0.9%) than the Surgicel plug technique (0%).

Endoclose closure with CT device has been studied many times; most of results were similar to ours. Elashry et al^6^ compared between 6 port-site closing techniques and showed that CT device was the second fastest technique and had the least complications. However, the sample size of CT device in his study was low (11 procedures). Lowry et al^7^ stated that facial closure with the CT device is fast and direct. Shetty et al^8^ study compared between hand closure and the CT device closure, sample size was 200 patients, 100 patients of each technique. The study showed that CT device was faster and resulted in less complications.^8^

Kim et al^2^ compared between CT device and EZ-close device on 78 patients, 39 patients each. They found that the EZ-close device was as safe as the CT device, however, EZ-close device was more efficient and enables the surgeon to be more self-sufficient while performing the closure. In terms of hernia rate, both resulted in 0% of port-site hernia; however, the low sample size makes it less reliable as the hernia rate in general is very rare. The time taken for the port-site closure with the EZ-close was faster than CT device. They attributed the less time taken in EZ-close device to the presence of suture thread within the device and to the less need for an assistance in some procedures. For the need of additional instrument, unlike CT group, there was no need for additional instrument in the EZ-close group.^2^ Some other authors also stated the necessity of using additional instruments with CT device.^9,15^

For the Surgicel plug, Moazin et al^13^ study which was done on 114 patients resulted in 1 case of hernia (0.8%) and 2 cases of non-infectious discharge (1.7%).^13^ There are some studies which performed similar techniques to the novel Surgicel plug prior to its invention. One study described the use of a bio-absorbable plug device to close the port-site and used it in 17 patients.^14^ Another one described the use of Surgicel^®^ for port-site closure by a roll-up and plug technique on 500 patients.^12^ Both studies resulted in a 0% rate of port-site related complications.^12,14^ Furthermore, in a retrospective comparative study which compared Vicryl sutures and Surgicel^®^ plugs, the authors stated that the Surgicel^®^ plug technique may offer some advantages over the Vicryl suture in terms of decreasing the overall cost, operative time, and iatrogenic bowel injuries caused by a closure device.^11^

In the current study, even though hernia rate is low, the higher rate of hernia in the novel plugging technique of this study when compared with similar studies of surgicel plug- might be attributed to the lack of subcutaneous tissue closure after plugging the ports with Surgicel. Therefore, subcutaneous tissue approximation by suture post Surgicel dipping might play a role in hernia prevention. Moreover, it was observed that one of the main causes of hernia of the novel plugging technique is that sometimes the surgeon dips the Surgicel too much and through it inside the abdomen instead of keeping it at the port site, therefore, we need to make sure after dipping the Surgicel that it is in place, not fallen inside.

### Study limitations

The groups were not from the same surgical department and the study is retrospective, therefore, prospective cohort study is recommended to be done on more identical groups of patients, and both techniques should be performed by one experienced surgeon.

In conclusion, the novel Surgicel plug technique is easy, safe, and effective. There is no significant difference in terms of complications between the novel Surgicel technique and the endoclose technique.
